# Mentoring, Task Sharing, and Community Outreach Through the TutoratPlus Approach: Increasing Use of Long-Acting Reversible Contraceptives in Senegal

**DOI:** 10.9745/GHSP-D-15-00328

**Published:** 2016-08-11

**Authors:** Babacar Gueye, Jennifer Wesson, Djimadoum Koumtingue, Sara Stratton, Claire Viadro, Hawa Talla, Etienne Dioh, Carol Cissé, Boniface Sebikali, Bocar Mamadou Daff

**Affiliations:** a IntraHealth International, Dakar, Senegal; b IntraHealth International, Chapel Hill, NC, USA; c Palladium, Washington, DC, USA; d Ministry of Health and Social Action, Division of Reproductive Health and Child Survival, Dakar, Senegal

## Abstract

**Background:**

To broaden access to family planning in rural areas and improve contraceptive prevalence, Senegal, in the context of wide method choice, is promoting implants and the intrauterine device, currently used throughout the country by only 5.6% of women of reproductive age who are in union, primarily urban women.

**Methods:**

The TutoratPlus performance improvement approach strengthens family planning clinical skills, particularly for long-acting reversible contraceptives (LARCs), through mentoring, task sharing, and community outreach. Following a 2013 baseline situation analysis, 290 participating facilities in 12 of Senegal's 14 regions developed action plans to address gaps identified in 3 areas: provider performance, equipment, and infrastructure. Between 2013 and 2014, 85 trained mentors coached, demonstrated skills, and observed 857 providers, including nurses, nonclinical family planning counselors, and community health workers (CHWs), in LARC service provision through two 5-day visits per facility at 21-day intervals. We used routine service delivery data and TutoratPlus mentoring data to assess changes in contraceptive use, including LARCs, 6 months before and 6 months after the mentoring intervention among 100 of the facilities with complete data.

**Results:**

The baseline assessment of 290 facilities found that fewer than half (47%) had a provider who could offer at least 1 LARC method, and 64% to 69% lacked kits. Post-intervention, all 290 facilities were adequately equipped and clinically able to offer LARCs. Among the 552 clinical providers, the percentage with acceptable LARC performance (at least 80% of observation checklist items correct) doubled from 32% to 67% over the 2 mentoring visits. In the 100 facilities with available comparison data, the number of new LARC users rose from 1,552 to 2,879 in the 6 months pre- and post-intervention—an 86% increase.

**Conclusion:**

Success of the TutoratPlus approach in Senegal is likely in part attributable to addressing facility-specific needs, using on-site mentoring to assess provider capacity, and achieving workplace enhancements through community engagement. Without CHW-initiated community outreach, LARC uptake might have been lower. Although task sharing requires institutionalization within national health systems, TutoratPlus demonstrates that provider skills can be improved, facilities can be better equipped, and demand can be promoted using existing government and community resources.

## Abstract (precis)

Mentoring, task sharing, and community outreach at 100 rural facilities in Senegal led to an 86% increase over 6 months in the number of women choosing long-acting reversible contraceptives (from 1,552 to 2,879). Concurrent improvement of facilities and provider skills, coupled with the application of Senegal’s task-sharing policy, are increasing the range of contraceptive methods available to women throughout the country.

## BACKGROUND AND SIGNIFICANCE

Despite concerted efforts by government, civil society, donors, and other stakeholders to address family planning unmet need, contraceptive prevalence in the West Africa region lags behind other parts of Africa and the world.^1^ As of 2013, contraceptive prevalence in Western Africa was 18% for any method and 12% for modern methods, versus 33% and 28%, respectively, in Eastern Africa.^2^ Given the large youth population in West Africa, the 83 million women of reproductive age estimated in need of family planning in 2015—up from 69 million in 2008^1^—will only increase.

At the same time, countries in the region suffer critical shortages of physicians, nurses, and midwives. They typically have fewer than 2 health professionals per 1,000 people, with those professionals overwhelmingly based in urban areas and higher-level facilities.^3^ Because roughly two-thirds (63%) of the population in sub-Saharan Africa is rural,^4^ many rural West Africans lack access to high-quality, facility-based family planning and other health services. Higher fertility and lower contraceptive use in rural areas of sub-Saharan Africa^5^^-^^8^ lead to more unwanted pregnancies and higher infant mortality^9^—indicating the need for greater access to good-quality services.

In Senegal, the modern contraceptive prevalence rate (mCPR) remains low, although it rose from 12% to 20% between 2010 and 2014.^10^ As part of commitments made during the 2012 London Summit on Family Planning,^11^ Senegal pledged to achieve a 27% CPR (by reaching an additional 350,000 women in union, including youth) and aspired to reduce unmet need from 30% to 15% by the end of 2015. As of the 2014 Continuous Demographic and Health Survey, unmet need had declined to 25%.^10^ To continue pursuing these aims, Senegal has put more emphasis on improving access to long-acting reversible contraceptives (LARCs) as part of a broader method mix that fosters access and choice.^12^ Expanding access is especially important because only 5.6% of women of reproductive age in union in Senegal currently use LARCs.^10^ Rural women in particular, have difficulty accessing the midwives and gynecologists who provide long-acting methods and who are primarily located in urban and more densely populated areas.^13^

Senegal has put more emphasis on improving access to LARCs as part of a broader method mix that fosters access and choice.

One way to increase access to LARCs in rural and underserved communities is through task sharing, defined as a process “where appropriate, tasks that are normally performed solely by highly qualified health workers are also undertaken by health workers who have had less training and have fewer qualifications.”^14^ A review of published research focusing on task sharing in maternal and reproductive health found that task sharing has the potential to increase service provision and can produce “equivalent health professional performance across cadres and patient outcomes.”^15^ However, task sharing in maternal and reproductive health requires adequate provider skills and supervision.^15^

West Africa has been slower than other regions to adopt task-sharing strategies, although some countries in the region are beginning to show an increased interest.^16^ In 2008, IntraHealth International/Senegal, through its Maternal, Neonatal and Child Health/Family Planning/Malaria project, supported by the U.S. Agency for International Development (USAID), began advocating to allow nurses to provide LARCs. Although it was largely untried in West Africa at that time, the approach held promise in Senegal where nurses are the principal family planning service providers in the lower-level primary health care facilities that serve the majority of the population. To test this approach, in 2009 IntraHealth designed and implemented a pilot performance improvement intervention called Tutorat (French for tutoring), which used on-the-job mentoring and skills reinforcement to train 407 nurses and midwives from 6 regions and 52 facilities in intrauterine device (IUD) and implant insertion and removal and infection prevention. Tutorat built on Learning for Performance,^17^ a proven performance improvement approach applied in a variety of national and local contexts.

Tutorat pioneered an on-site methodology to seamlessly integrate training, supervision, follow-up, and facility-wide performance improvement while building local ownership and a sustainable culture of quality. Based on the promising and well-received results from the Tutorat pilot, the Ministry of Health and Social Action added task sharing to Senegal’s reproductive health policies, norms, and protocols and committed to scale up task sharing nationwide. In 2011, IntraHealth’s USAID-funded Health Services Improvement Project developed the enhanced TutoratPlus performance improvement approach. TutoratPlus mobilizes all essential actors (providers, supervisors, managers, community leaders, and clients) to address service delivery shortfalls by strengthening the work environment and health worker skills and competencies using mentorship and community mobilization. One aim is to broaden the availability of family planning services, including LARCs, in all lower-level health facilities through task sharing. As a comprehensive intervention, TutoratPlus also addresses a number of other areas, but these are not the focus of this analysis. This article describes the multipronged Tutorat-Plus efforts in the area of family planning and examines LARC acceptance before and after implementation of TutoratPlus in lower-level health facilities. Our description of a nationally scaled-up task-sharing intervention will be of interest to other countries considering similar interventions, especially in the subregion of West Africa.

TutoratPlus mobilizes all essential actors, from providers, supervisors, and managers to community leaders and clients, to address service delivery shortfalls.

## PROGRAM DESCRIPTION

TutoratPlus is a problem-solving performance improvement approach designed to achieve high-quality delivery of high-impact maternal, neonatal, and child health services, including family planning, by mentoring health workers in their facilities and strengthening the environments in which they work (Box 1). TutoratPlus developed 6 mentoring packages covering family planning; maternal health; disease management, including integrated management of childhood illnesses; facility management and organization of services (logistics, human resources, and financial systems); monitoring and evaluation; and health promotion and demand promotion. Implementation of TutoratPlus in Senegal has unfolded through 4 steps: situation analysis of service delivery gaps, development of facility- and district-level action plans, identification and training of mentors, and worksite coaching and supervision. The focus of this article is on the family planning mentoring package and its results; therefore, all references to TutoratPlus in general terms should be understood as referring to the family planning mentoring component.

BOX 1. **TutoratPlus Principles**

TutoratPlus is based on several principles:

- Classical trainings generally do not provide enough practical experience and also take providers away from their posts. The TutoratPlus on-site mentoring approach was developed in response to these challenges.
- Improving provider competencies is not enough to ensure high-quality services. The provider’s work environment must also be taken into account, including infrastructure, equipment, and supplies. TutoratPlus examines the environment through a situation analysis.
- The contents of provider training and mentoring should be based on an empirical identification of performance gaps. Every TutoratPlus mentor is equipped with tools to measure and evaluate performance.
- Ensuring high-quality services is a whole-system process. TutoratPlus includes local officials and health committees in creating action plans and evaluating progress at regular review meetings.

At the facility level, the TutoratPlus approach has been implemented among qualified nurses and midwives with clinical skills who provide family planning and other health services, as well as nonclinical and community health workers who provide family planning counseling and conduct demand promotion activities. All activities are carried out by existing ministry staff, with technical support from IntraHealth. The mentoring component, which is ongoing, started in October 2013.

**Figure:**
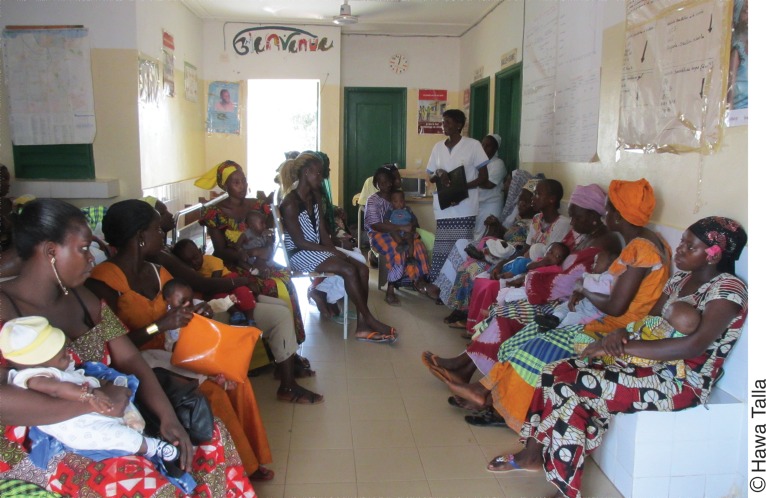
A provider counsels a group of women about family planning at a health post in Ziguinchor, Senegal.

### Analysis of Service Delivery Gaps

The Ministry of Health and Social Action and IntraHealth/Senegal conducted a baseline situation analysis in all facilities before the intervention. The assessment examined:

- Providers’ family planning knowledge and skills
- The work environment (e.g., availability of running water, electricity, infection prevention materials, and IUD and implant insertion kits)
- Health facility infrastructure
- Work flow, management, and supervision
- Relationships with the community

**Figure:**
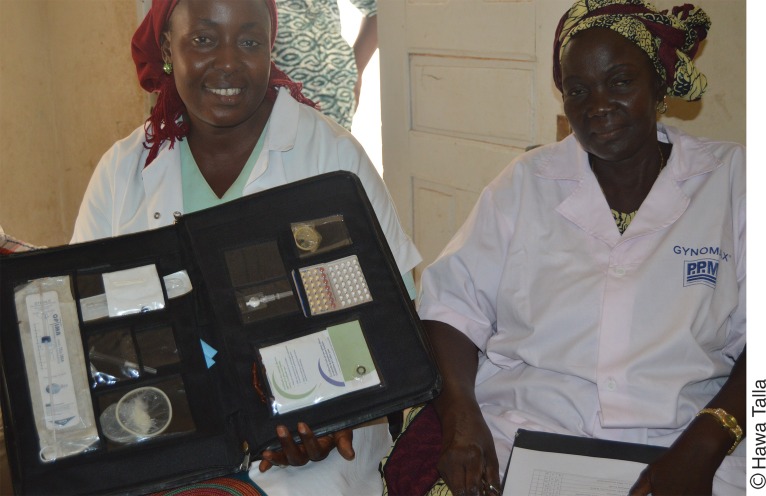
A family planning provider with her mentor at the Mbouma Fatick health post in Senegal displays the job aid she uses to counsel clients on the complete range of contraceptive methods from which they can choose.

We focused on identifying gaps contributing to poor performance.

### Development of Action Plans

Each facility developed an action plan to address gaps identified in the situation analysis pertaining to the work environment and provider skills. Facility managers and community members developed the facility-level action plans. Districts then prepared a broader district action plan that synthesized the facility-level plans. The district-level action plans outlined activities to address 3 primary gap areas: provider performance, equipment (such as infection prevention materials and LARC insertion and removal kits), and infrastructure improvements to help facilities maintain client privacy. The district-level action plans also identified needed resources, the individuals responsible for each activity, and the contributions and commitments of local health committees, local authorities, the government, and other partners. In all, 1,330 facilities in Senegal’s 14 regions participated in this process and developed action plans (Box 2). Among them, 290 facilities in 12 regions included strengthening of family planning services in their action plans. These 290 facilities are the subject of this article.

The district-level action plans addressed 3 gap areas: provider performance, equipment (such as infection prevention materials and LARC kits), and infrastructure improvements to maintain client privacy.

BOX 2. **Evidence-Based Scale-Up of TutoratPlus**

The Ministry of Health and Social Action, with the support of IntraHealth, continues to scale up the TutoratPlus approach. As of 2015, 1,330 health facilities were enrolled in the TutoratPlus program, a level of expansion made possible because of the ministry’s strong support for task sharing and decentralization of services such as provision of long-acting reversible contraceptives. Demonstrating its commitment to evidence-based decision making, the ministry’s initial decision to scale up TutoratPlus was based on the evidence generated by the 2009 pilot Tutorat in 52 facilities.

### Identification and Training of Family Planning Mentors

District staff of the Ministry of Health and Social Action recruited mentors already working in the public-sector health system at the district level. The work with TutoratPlus did not constitute a new, full-time job but rather was a supplemental duty in addition to mentors’ scope of work, with no extra salary provided. The positions were advertised; qualifications included current competency in family planning provision, including LARCs, at least 3 years’ professional experience, an interest in serving as a mentor on a longer-term basis, and strong interpersonal communication skills.

Ministry staff selected 85 family planning mentors from among district-level staff in 56 districts, with each district choosing 1 or 2 of the most highly qualified candidates (depending on district size) after carefully assessing applicants’ knowledge and skills. Mentors were selected from sites with more than 1 service provider, so that service provision at their own facilities would not stop when the mentors made coaching visits to other facilities. In addition, district health teams jointly scheduled the visits with mentors and were responsible for ensuring that the mentor’s absence from the facility did not have a negative impact on service delivery. A team composed of ministry and IntraHealth trainers conducted the 10-day mentor training. The training covered principles of adult learning and communication and coaching skills, as well as practical supervision of providers in the workplace. While the mentors were required to possess the requisite clinical skills, the training included a refresher of clinical knowledge, especially with regard to mentoring other providers. All mentors also participated in test mentoring in nearby sites, and each mentor received an extensive manual on how to conduct the mentoring.

### Worksite Mentoring and Supervision

On average, each mentor was responsible for visiting providers in 4 lower-level facilities, and mentors conducted two 5-day visits at each facility with a 21-day interval between visits. As noted previously, district health teams provided support to the mentors in scheduling and organizing their visits. All providers in each facility were included in the mentoring and supervision, including community health workers. During the first visit, armed with information from the situation analysis about service delivery gaps, the mentors met with local officials, health committee members, and facility staff to share and validate the identified gaps, as well as the intervention objectives and activity plans. During these visits, the mentors also individually observed family planning providers to assess their LARC skills with anatomic models. Following these observations, the mentors gave feedback to providers and performed a demonstration to correct shortcomings. When family planning providers did not achieve the required performance level (at least 80% of items correct on a standardized observation checklist^18^ from the World Health Organization [WHO] and approved by the Ministry of Health and Social Action) during the first observation, the mentors asked them to carry out repeat demonstrations as many times as needed to master the skill. On average, providers achieved an acceptable performance level after 5 anatomic model demonstrations. After providers demonstrated acceptable skills with the models, they applied the skills with actual clients under mentor supervision.

During the second visit approximately 3 weeks later, the mentors used the same observation checklist to assess providers’ mastery of LARC clinical skills and address any lingering performance gaps.^18^ On the last day of the visit, the mentors presented their findings on the outcomes of the mentorship process to all of the partners involved in implementation, helping to emphasize the importance of this approach and build ownership. Subsequent to the second visit, mentors also shared their results with the district teams responsible for follow-up, supervision, and advocacy with local groups to assume responsibility for remaining equipment and infrastructure gaps. Mentors referred providers who did not meet the criteria for acceptable skills to district supervisors, who continued to mentor them until they met standards.

## METHODOLOGY

The results for this paper are drawn from 4 data sources:

1. **The baseline situation analysis** provided data on availability of family planning services, including LARCs and insertion and removal kits for implants and IUDs.
2. **An internal IntraHealth database** produced data regarding TutoratPlus for each health facility receiving family planning mentoring and worksite support; financial and in-kind contributions of health committees and local constituencies were also recorded in this database.
3. **The standardized observation checklist,** based upon WHO guidance and included in the official family planning curriculum, generated data on provider performance during the mentoring visits.^18^
4. **Service delivery data** were extracted from the Data Exchange System (DES), the ministry’s automated information system that uses mobile phones and the Internet to transfer and share information related to reproductive health,^19^ such as the number of new family planning users, the number of antenatal care visits, and the number of assisted deliveries. At the end of each month, data are sent from the health facility to the district via mobile phone. After validation, the data become visible to anyone accessing the system.

**FIGURE 1.**
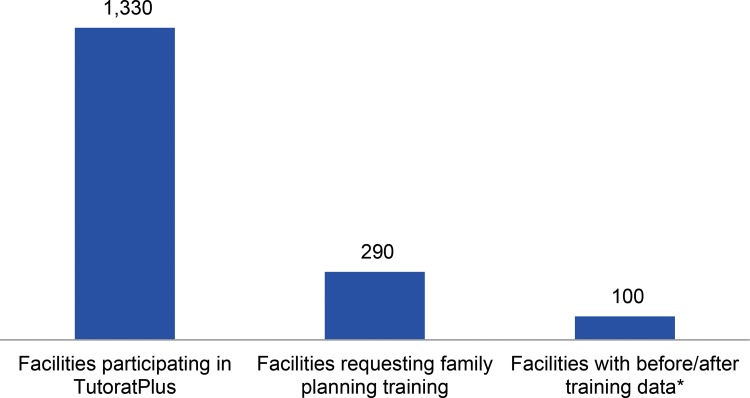
Number of Facilities Participating in TutoratPlus and Included in the Analysis, Senegal, 2013–2014 * Complete data were available for 100 of the 290 health facilities; thus, only the subset of 100 facilities was included in the comparison analysis.

To assess changes in use of family planning, including LARCs, we merged the DES and internal databases to match the date of TutoratPlus mentoring with the number of family planning users served. For each facility, we constructed “before” and “after” periods, covering the 6 months before and 6 months after the TutoratPlus mentoring intervention in that facility. Thus, every facility has different dates comprising their before-and-after periods. For example, the comparison for a health facility that received mentoring in June 2014 would be between the 2 time periods of December 2013–May 2014 (before) and June 2014–November 2014 (after). Complete data were available for 100 of the 290 health facilities; thus, only a subset of 100 facilities is included in the comparison analysis (Figure 1). We excluded the remaining 190 facilities from the comparison analysis because they either lacked complete before-and-after data (n = 167) or had no data at all (n = 23) in the DES. The 2 samples (i.e., the full sample of 290 facilities and the subsample of 100 facilities) shared many characteristics, including similar distribution among types of structures, average number of health workers, region, and urban/rural setting. For other indicators, such as the achievement of acceptable performance standards, we report results for all 290 facilities.

## RESULTS

**FIGURE 2.**
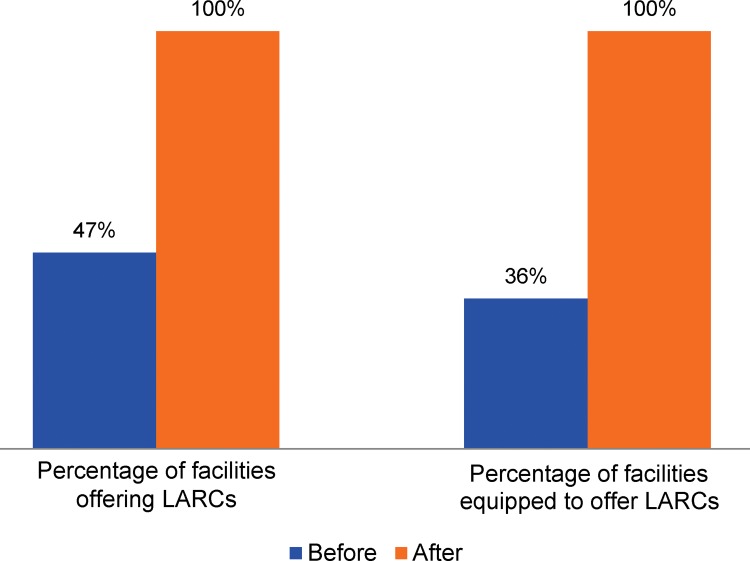
Percentage of Facilities Equipped to Offer LARCs and With a Provider Able to Offer LARCs, Before and After TutoratPlus (N=290) Abbreviation: LARCs, long‐acting reversible contraceptives.

The 2013 situation analysis of all 290 facilities found that fewer than half (47%) had a provider who could offer at least 1 long-acting reversible method, and only 36% had at least 1 IUD or implant insertion and removal kit immediately available (Figure 2). Sixty-four percent of facilities did not have implant kits in stock and 69% lacked IUD kits. After the workplace and mentor interventions and community engagement, all 290 facilities had the supplies to offer LARCs to their clients (Figure 2).

After the workplace and mentor interventions and community engagement, all 290 facilities were adequately equipped with the supplies to offer LARCs to their clients.

Of the 857 providers trained as part of the TutoratPlus intervention in the 290 facilities, 552 were clinical staff (the vast majority of whom were nurses and midwives) and 305 were nonclinical providers, such as community health workers, who received mentoring solely on family planning counseling of new clients. During the period beginning with the first mentor visit and ending with the second visit, providers carried out an average of 5 LARC insertions per facility. The proportion of mentored providers who had an acceptable performance level (at least 80% of observation checklist items correct) increased from 32% to 67% among the 552 clinical providers after the facility-based mentoring.

Together, health committees and local authorities contributed an estimated 67,023,288 CFA francs (FCFA) or roughly US$114,570. Examples of the kind of support received by facilities include: infection prevention supplies, insertion and removal kits, and support for structural improvements to the rooms where family planning counseling and/or family planning services are provided, which improved the confidentiality of family planning services.

**FIGURE 3.**
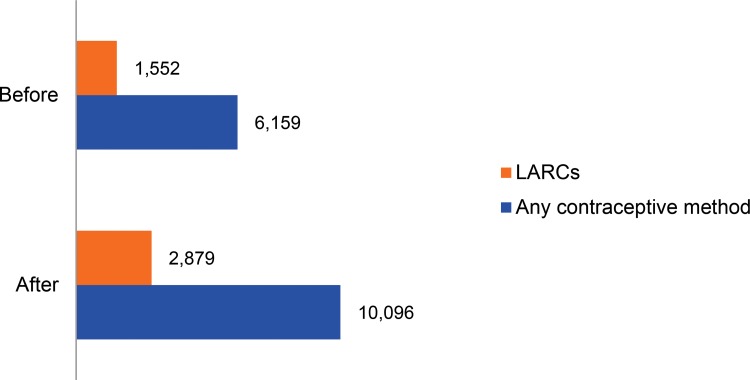
Number of New Contraceptive Users (of Any Method and of LARCs Specifically), 6 Months Before and 6 Months After TutoratPlus (N=100 Facilities) Abbreviation: LARCs, long‐acting reversible contraceptives.

In the subset of 100 facilities for which comparison data on family planning service delivery were available, the number of new clients receiving any family planning method increased by 64% across the two 6-month time periods before and after the mentor intervention. The number of new LARC users increased by 86%, or 1,327 users (Figure 3). Within the category of LARC users, the distribution of implant versus IUD users did not change significantly across the 2 time periods. In the 6 months before TutoratPlus, 83% of LARC users adopted the implant and 17% the IUD. In the 6 months following TutoratPlus, 87% of LARC users were implant users and 13% were IUD users (not shown).

The number of new LARC users increased by 86% after the mentoring intervention.

## DISCUSSION

Senegal has made significant family planning progress. From 2010 to 2014, the mCPR rose by 8 percentage points, from 12% to 20%, among women of reproductive age in union.^10^ In this short period of time, the country made as much progress as in the previous 20-year period (1992–2011). Substantially more of this recent increase occurred in rural areas, where the mCPR rose by 86% (from 7% to 13%), compared with a 45% increase in the urban mCPR (from 20% to 29%).^10^^,^^20^ The broadening of the family planning method mix through expanded access to LARCs in rural areas undoubtedly contributed to this accelerated progress, with a 230% increase in LARC use, from 1.7% in 2010 to 5.6% in 2014.

The principal barrier that TutoratPlus sought to address in its family planning work was the lack of access to good-quality LARC services in rural and underserved areas. Within the wider context of rising acceptance of family planning, the sharp increase in the number of women adopting LARCs after implementation of provider mentoring and structural improvements demonstrates that there was a latent demand for these methods. Before the task-sharing policy was instituted and applied via TutoratPlus, only physicians and midwives—who are based at more centralized and higher-level facilities—could provide implants and IUDs. Previously, there was less chance for rural women, in particular, to adopt a LARC even if they knew about long-acting methods, because not all lower-level facilities offered LARCs, and travel to higher-level facilities is often not possible.

Some of the achievements of the TutoratPlus approach can be attributed to the fact that it addressed the performance and quality-of-care needs of each district and facility, based on evidence gathered during the baseline situation analysis. Based on previous experience with capacity building, the TutoratPlus approach acknowledges that not only family planning providers exhibit gaps in clinical and counseling skills; health facilities can also have gaps, notably in infrastructure and equipment, and communities must be informed to promote demand. This broader and more holistic approach enabled district stakeholders to create action plans based on actual needs rather than by trying to address LARC provision with the same approach for all facilities.

By openly presenting the results of the baseline situation analysis to health committees and local authorities, TutoratPlus encouraged these community stakeholders to get involved in extending and improving services in their local health facilities. As a result of this engagement, communities invested their own money in the health facilities (for example, channeling resources toward staff recruitment, improvements to counseling rooms, and acquisition of equipment and infection prevention materials), thereby providing some measure of sustainability for family planning and reproductive health services. In addition to the mentors’ activities, the vital role of the health committees and local authorities in strengthening the facilities’ work environment likely contributed to the increase in the number of women choosing LARCs.

Communities invested their own money in the health facilities.

Another key feature of the TutoratPlus design is that it seeks to address the difficulties associated with formal, off-site trainings. The latter are often expensive; remove providers from their facilities for several days or even weeks; and provide insufficient practical training with actual clients. The on-site mentoring approach adopted by TutoratPlus allowed for training based on a mentor’s assessment of an individual provider’s capacity in their work environment. Providers also had the opportunity to practice their skills with actual clients while under mentor supervision. Moreover, each mentor offered training and support to all the providers (both clinical and community health personnel) in a facility, increasing the efficiency of the approach.

The community health workers, in turn, informed their communities about the new LARC services available at their health facilities, in addition to the other family planning methods already routinely available. Without this element of demand promotion, LARC uptake rates probably would not have been nearly as high.

TutoratPlus is currently operating in 12 of Senegal’s 14 regions, supported by IntraHealth’s USAID-funded Health Services Improvement Project. The Ministry of Health and Social Action has embraced the approach and is currently working with the various branches of the ministry to integrate it into district and regional work plans. Once TutoratPlus is written into work plans, other partners supporting the ministry will be expected to adopt the approach as well, resulting in an institutionalization of TutoratPlus.

The number of new users served with a family planning method, including LARCs, may appear to be small in comparison with the national goal of serving 250,000 new family planning users in public facilities and at the community level over a 4-year period (2012–2015).^21^ However, the fact that 290 facilities, of the 1,330 total facilities participating in TutoratPlus, served over 10,000 new users in a 6-month period demonstrates that TutoratPlus will make a substantial contribution toward Senegal achieving its ambitious goal.

Beyond the investments made by community-based entities such as the health committees and local authorities, there are other steps that should be taken to ensure sustainability and institutionalization of both TutoratPlus and the task-sharing approach for LARCs.

1. The Ministry of Health and Social Action intends to fully disseminate the national policies and protocols, ensuring a favorable climate for task sharing and ensuring that providers know the policy.
2. The skills needed to provide LARCs should be integrated into nurses’ in-service training in a flexible manner. Factors such as provider workload and client demand will shape decisions about which nurses are most likely to make regular use of LARC skills.
3. Although TutoratPlus along with health committees and local authorities took steps to provide needed IUD and implant insertion and removal kits and equipment, some health facilities still face shortages. It is vital that the national logistics system stock and deliver this equipment in a timely manner to ensure that LARCs remain available.
4. The ministry is working to ensure continued regular access to family planning commodities. The rate of stock-outs in Senegal decreased dramatically (to less than 2%) after the introduction of the “Informed Push Model.”^22^ If commodities are not available, women will not be served.
5. The mentors themselves need ongoing support, including supervision of their work as mentors. Because mentors spend time away from their own district-level facilities to mentor and supervise lower-level providers, ongoing provisions must be made through adequate staffing to support the mentors’ facilities while they are away.
6. The Ministry of Health and Social Action should consider the mentors a valuable resource and make efforts to retain them in the districts and within the public health system. Over the course of the 2-year TutoratPlus intervention period, roughly half of the mentors who were trained left their facilities of origin. This necessitated training new mentors, and also meant that there were gaps in the support available to family planning providers offering LARCs.

### Limitations

The pre/post-intervention study design and the absence of a control group are limitations of the analysis. We chose the pre/post-intervention design because the ministry mandated scaling up the TutoratPlus approach in all 1,330 health facilities covered by IntraHealth’s Health Services Improvement Project; thus, a control group was not feasible. A second limitation pertains to the lack of routine data of adequate quality for many of the health facilities that participated in TutoratPlus for family planning and for a longer period of time. It is possible that the 100 facilities with complete data are functioning at a higher level (at least with regard to recordkeeping) than those without complete data, which would introduce a bias. The 2 samples (i.e., the 290 facilities and the subsample of 100 facilities) were similar for many basic characteristics; however, it was not possible to more fully examine the reasons for incomplete data.

## CONCLUSIONS

The experience with TutoratPlus demonstrated that when good-quality services are available and women are informed of their choice of contraceptive methods, including LARCs, more women choose to use a LARC. The approach also showed how capacity can be built and work environments improved using existing district-level and community resources, while promoting parallel demand for services. These concurrent facility-level and provider-level improvements, coupled with the application of Senegal’s task-sharing policy, are increasing the range of contraceptive methods available to women throughout the country. As LARCs continue to become more available and acceptable, the mCPR is likely to increase, thereby contributing to the achievement of the government’s ambitious goals and to the commitments established through the Ouagadougou Partnership^23^ and the Family Planning 2020 partnership.^11^

Although the Ministry of Health and Social Action modified its reproductive health policies, norms, and protocols to incorporate task sharing and authorize nurses to provide LARCs, the task-sharing approach requires an even stronger strategy to scale up this approach and achieve institutionalization within the national health system. As a step in this direction, the ministry and IntraHealth are conducting a national evaluation of TutoratPlus under the guidance of a ministry-led committee charged with articulating a strategy to maximize the approach’s application within the public-sector health system. National-level institutionalization and the evaluation results may allow the approach to be assessed as a best practice and eventually rolled out throughout the West Africa region.

## Notes

**Peer Reviewed**

## References

[1] Gribble J Family planning in West Africa [Internet]. Washington (DC): Population Reference Bureau; 2008 Mar [cited 2015 Oct 5]. Available from: http://www.prb.org/Publications/Articles/2008/westafricafamilyplanning.aspx

[2] Population Reference Bureau (PRB). Family planning worldwide 2013 data sheet. Washington (DC): PRB; 2013 Available from: http://www.prb.org/pdf13/family-planning-2013-datasheet_eng.pdf

[3] World Health Organization (WHO). The world health report 2006: working together for health. Geneva: WHO; 2006 Available from: http://www.who.int/whr/2006/en/

[4] World Bank. Agriculture & rural development [Internet]. Washington (DC): World Bank; c2016 [cited 2015 Oct 5]. Available from: http://data.worldbank.org/topic/agriculture-and-rural-development

[5] Central Statistical Agency [Ethiopia]. Ethiopia mini demographic and health survey 2014. Addis Ababa (Ethiopia): Central Statistical Agency; 2014 Available from: http://www.unicef.org/ethiopia/Mini_DHS_2014__Final_Report.pdf

[6] Ghana Statistical Service (GSS); Ghana Health Service (GHS); ICF International. Ghana demographic and health survey 2014. Rockville (MD): ICF International; 2015 Co-published by GSS and GHS. Available from: https://dhsprogram.com/pubs/pdf/FR307/FR307.pdf

[7] Kenya National Bureau of Statistics (KNBS); Ministry of Health (MOH); National AIDS Control Council (NACC); Kenya Medical Research Institute (KMRI); National Council for Population and Development (NCPD); ICF International. Kenya demographic and health survey 2014. Nairobi (Kenya): KNBS; 2015 Co-published by MOH, NACC, KMRI, NCPD, and ICF International. Available from: https://dhsprogram.com/pubs/pdf/FR308/FR308.pdf

[8] National Population Commission (NPC) [Nigeria]; ICF International. Nigeria demographic and health survey 2013. Abuja (Nigeria): NPC; 2014 Co-published by ICF International. Available from: https://dhsprogram.com/pubs/pdf/FR293/FR293.pdf

[9] Rutstein SO. Effects of preceding birth intervals on neonatal, infant and under-five years mortality and nutritional status in developing countries: evidence from the Demographic and Health Surveys. Int J Gynaecol Obstet. 2005;89 Suppl 1:S7–S24. 10.1016/j.ijgo.2004.11.012. 15820369

[10] Agence Nationale de la Statistique et de la Démographie (ANSD); ICF International. Sénégal: enquête démographique et de santé continue (EDS-continue) 2014. Dakar (Senegal): ANSD; 2015 Co-published by ICF International. Available from: http://dhsprogram.com/pubs/pdf/FR305/FR305.pdf

[11] Family Planning 2020. About us: Family Planning 2020 [Internet]. Washington (DC): Family Planning 2020; c2015 [cited 2015 Oct 5]. Available from: http://www.familyplanning2020.org/about

[12] Population Council. Long-acting reversible contraception in the context of full access, full choice. New York: Bellagio Group on LARCs; 2013 Available from: http://www.popcouncil.org/news/long-acting-reversible-contraception-in-the-context-of-full-access-full-cho

[13] Ministère de la Santé et de l’Action Social. Plan national de développement sanitaire 2009-2018. Dakar (Senegal): Ministère de la Santé et de l’Action Social; 2009.

[14] International Planned Parenthood Federation. IMAP statement on task sharing in sexual and reproductive health. IPPF Med Bull; 2013 Jan. Available from: http://www.ippf.org/sites/default/files/imap_statement_task_sharing.pdf

[15] Dawson AJ Buchan J Duffield C Homer CSE Wijewardena K. Task shifting and sharing in maternal and reproductive health in low-income countries: a narrative synthesis of current evidence. Health Policy Plan. 2014;29(3):396–408. 10.1093/heapol/czt026. 23656700

[16] Konaté MK Maiga M McGinn E Chen A Repositioning family planning in West Africa: task sharing synthesis report. Washington (DC): Futures Group, Health Policy Project; 2015 Available from: http://www.healthpolicyproject.com/pubs/1879_TaskSharingSynthesisNov.pdf

[17] IntraHealth International. Learning for performance: a guide and toolkit for health worker training and education programs [Internet]. Chapel Hill (NC): IntraHealth International; 2008 Jan [cited 2015 Dec 21]. Available from: http://www.intrahealth.org/lfp/

[18] Département Santé et Recherche génésiques de l’Organisation mondiale de la Santé (OMS/RHR); l’Ecole de santé publique de l’Université Johns Hopkins/Centre pour les programmes de communication (CCP), projet Knowledge for Health. Planification familiale: un manuel a l’intention des prestataires de services du monde entier. Baltimore: CCP; 2011 Co-published by OMS. Available from: http://www.who.int/reproductivehealth/publications/family_planning/9780978856304/fr/

[19] Potenziani D SEDA automated health data exchange system: applying mHealth to improve data monitoring in Senegal. : Levine R Corbacio A Konopka S Saya U Gilmartin C Paradis J, mHealth Compendium, Volume 5. Arlington (VA): African Strategies for Health, Management Sciences for Health; 2015 p. 40–41. Available from: https://www.msh.org/sites/msh.org/files/2015_08_msh_mhealth_compendium_volume5.pdf

[20] Agence Nationale de la Statistique et de la Démographie (ANSD) [Sénégal]; ICF International. Enquête démographique et de santé à indicateurs multiples au Sénégal (EDS-MICS) 2010-2011. Calverton (MD): ICF International; 2012 Co-published by ANSD. Available from: http://www.dhsprogram.com/pubs/pdf/FR258/FR258.pdf

[21] Ministère de la Santé et de l’Action Social. Plan d’action national de planification familiale 2012-2015. Dakar (Senegal): Ministère de la Santé et de l’Action Social; 2012.

[22] Daff BM Seck C Belkhayat H Sutton P. Informed push distribution of contraceptives in Senegal reduces stockouts and improves quality of family planning services. Glob Health Sci Pract. 2014;2(2):245–252. 10.9745/GHSP-D-13-00171. 25276582 PMC4168620

[23] Population Reference Bureau (PRB). Family planning: Francophone West Africa on the move—a call to action. Washington (DC): PRB; [2011]. Available from: http://www.prb.org/pdf12/ouagadougou-partnership_en.pdf

